# Single-shot hard X-ray spectrometer with uniform spatial and spectral response

**DOI:** 10.1107/S1600577526007599

**Published:** 2026-08-26

**Authors:** Madison M. Singleton, Peihao Sun, Lex Andersen, Karen Appel, Adrien Descamps, Siegfried H. Glenzer, Sebastian Göde, Oliver S. Humphries, Naresh Kujala, Emma E. McBride, Thomas R. Preston, Sripati V. Rahul, Ulf Zastrau, J. B. Hastings, Luke B. Fletcher

**Affiliations:** a SLAC National Accelerator Laboratory Menlo Park CA94025 USA; b Dipartimento di Fisica e Astronomia Università degli Studi di Padova 35131Padova Italy; c European XFEL GmbH 22869Schenefeld Germany; d School of Mathematics and Physics Queen’s University Belfast University Road BelfastBT7 1NN United Kingdom; ESRF – The European Synchrotron, France

**Keywords:** high-resolution spectrometers, stochastic correlation X-ray spectroscopy, glassy carbon, single-shot spectrum, SASE, XFELs

## Abstract

Knowledge of the shot-to-shot incident spectrum in X-ray free-electron laser (XFEL) experiments is crucial for the analysis and interpretation of experimental data collected from various spectroscopic techniques such as X-ray Thomson scattering, resonant inelastic X-ray scattering and X-ray pump–probe experiments. In this article, we present a novel spectrometer design capable of accurately measuring the incident self-amplified spontaneous emission (SASE) XFEL spectrum on a shot-to-shot basis that is free of spatial and spectral inhomogeneities, such as chirp and central energy jitter that are inherent to XFEL radiation. The setup consists of glassy carbon as a dispersive element, a flat Si (444) analyzer crystal and a 2D pixel detector. Direct comparisons with existing spectral monitors are obtained using a reconstruction method based on frequency-resolved stochastic correlations between the measured incident SASE spectrum and the well characterized collective scattering excitations from aluminium.

## Abstract 2

We demonstrate a single-shot hard X-ray spectrometer based on small-angle scattering from glassy carbon that is insensitive to X-ray free-electron laser beam-dependent spatial and spectral variations. Comparison with established X-ray Thomson scattering diagnostics using stochastic correlation X-ray spectroscopy demonstrates full-bandwidth coverage with millielectronvolt-scale spectral resolution, enabling reliable single-shot spectroscopic measurements.

## 1. Introduction

Self-amplified spontaneous emission (SASE) is the dominant mechanism for X-ray generation in X-ray free-electron lasers (XFELs). Electron bunches are produced through photoemission, a process that introduces intrinsic random noise in the initial phase-space distribution. These fluctuations seed the FEL instability and are exponentially amplified through the electron-radiation interaction in the undulator, giving rise to strong shot-to-shot stochastic variations in the X-ray pulse structure. Because the longitudinal coherence length of the radiation is shorter than the electron-bunch duration, a typical SASE pulse consists of many independent longitudinal modes, resulting in a spiky temporal and spectral profile and corresponding fluctuations in the spectral bandwidth on a shot-by-shot basis. In addition, fluctuations in the electron-beam energy induce jitter in the central photon energy. Precise single-shot characterization of the SASE spectrum is therefore required for accurate spectroscopic measurements.

To measure these shot-to-shot variations, XFEL facilities commonly use dispersive X-ray spectrometers that map photon energy onto a position-sensitive detector through Bragg diffraction from a crystal. Bent-crystal analyzers are regularly used, as they provide high spectral resolution over a large range and single-shot capability. However, these instruments are sensitive to spatial–spectral chirp and jitter of the beam’s central energy, introducing systematic errors when transverse spatial fluctuations are mapped onto the measured spectrum. In a bent-crystal spectrometer, the crystal maps different sections of the beam’s spatial position to energy channels leading to a distortion in the resulting spectrum (Zhu *et al.*, 2012 ▸). For instance, if a localized region of elevated intensity occurs in the lower portion of the X-ray beam, this portion is reflected to a corresponding position on the detector, with the resolution determined by the crystal’s radius of curvature, thickness and elastic constants (Kaganer *et al.*, 2021 ▸). Since the detector’s pixel position is calibrated to a single photon energy, the area will appear as a large energy spike in the spectrum, falsely recording a spatial fluctuation as a spectral feature. Moreover, the transverse spatial profile of a SASE FEL pulse at fixed photon energy exhibits significant shot-to-shot fluctuations arising from the random superposition of higher-order modes (Sun *et al.*, 2019 ▸). These higher-order modes introduce uncertainty and instability in diagnostics that relate spatial and spectral information, therefore negatively affecting energy resolution (Sun *et al.*, 2019 ▸).

In this work, we present a simple spectrometer design that enables complete single-shot measurement of the incident SASE pulse that is free from effects caused by spatial chirp, central energy fluctuations, and inhomogeneities in the beam profile. The main concept is to insert a scatterer into the beam to create an angular dispersion, which is then converted by a flat analyzer crystal into an energy dispersion. With the analyzer placed sufficiently far away, the measured spectrum is insensitive to the beam’s spatial profile. We use vitreous (glassy) carbon, an amorphous microstructured scatterer with a large, nearly uniform, scattering cross section at low momentum transfers (Zhang *et al.*, 2010 ▸).

The integrated signal from all beam regions can thus be accurately binned, yielding a reconstructed energy spectrum representative of the full main beam.

## 2. Methods and instrumentation

### 2.1. Proof-of-concept experiment

The spectrometer was demonstrated at the High Energy Density Science beamline (HED) at the European X-ray Free Electron Laser (EuXFEL) facility (Zastrau *et al.*, 2021 ▸). The European XFEL creates X-rays in a bunch ‘train’, with up to 27000 pulses per bunch, delivered at megahertz rates (Zastrau *et al.*, 2021 ▸). To characterize the incident XFEL spectrum, an 8.31 keV SASE beam is first measured by a bent-crystal high-resolution hard X-ray single-shot (HIREX-II) spectrometer (Kujala *et al.*, 2020 ▸, 2026 ▸), followed by interaction with the sample and the resulting inelastic X-ray scattering [specifically, X-ray Thomson scattering (XRTS) in the plasma regime] collected using both a collective (forward scattering) and a non-collective (backward scattering) spectrometer. The beam transmitted through the sample passes through a 2 mm-thick glassy carbon sample, which angularly disperses the SASE beam.

**Figure 1:**
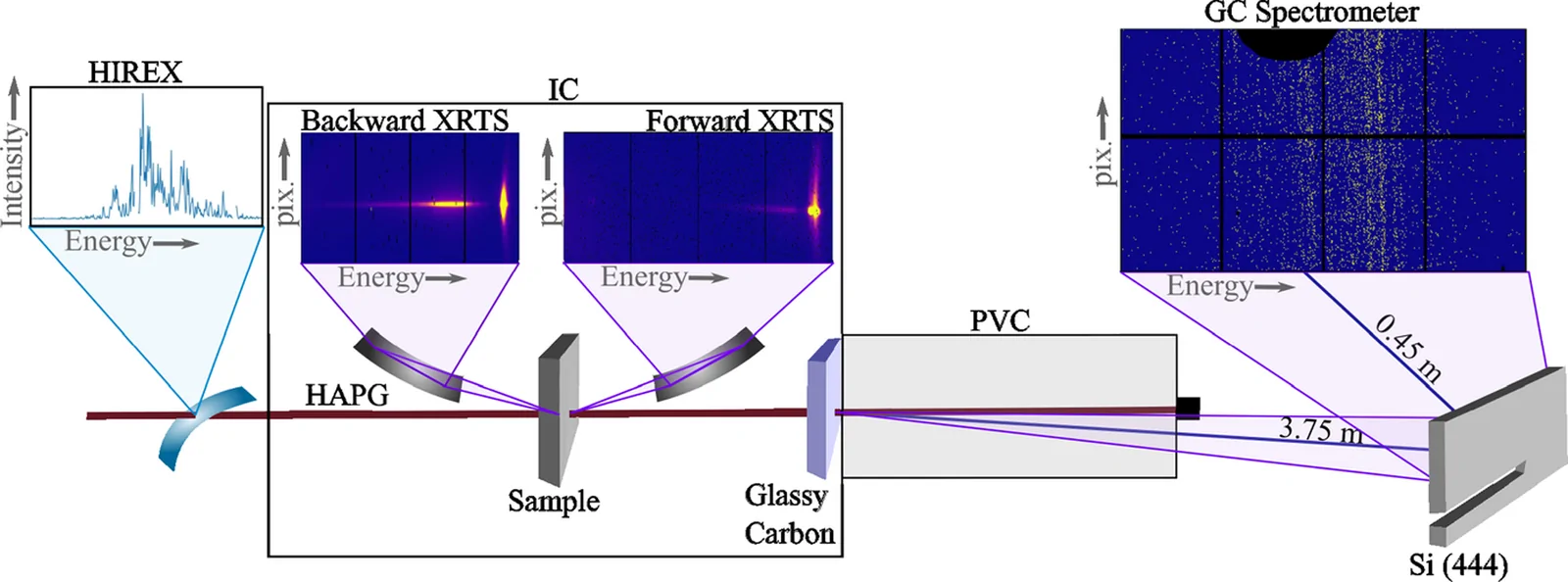
Schematic drawing of the experimental setup at the HED beamline of the European XFEL (not to scale). The incident X-ray spectrum is measured shot-by-shot using a high-resolution (HIREX) spectrometer. X-ray Thomson scattering (XRTS) from the sample is collected by von Hamos X-ray spectrometers based on HAPG crystals in both backward- and forward-scattering geometries within the interaction chamber (IC). A glassy carbon plate downstream of the sample provides the scattering signal, which is dispersed by a Si (444) flat analyzer crystal and recorded on a Jungfrau detector. A PVC pipe is used to transport the diffracted beam (in vacuum) from the glassy carbon to the Si crystal. The Si crystal’s design includes a notch, as pictured, for strain relief. The detector edges and beamstop are masked.

This angular dispersion is converted into spatial positions on the detector by Bragg reflection from a symmetric Si (444) flat crystal (8 cm × 4 cm), where the (444) reflection provides high angular dispersion and energy resolution near backscattering. A schematic drawing of the full setup is given in Fig. 1 ▸.

The HIREX spectrometer captures single-shot images of an incident SASE spectrum with a resolution of Δ*E*/*E* = 10^−4^ (Kujala *et al.*, 2020 ▸, 2026 ▸). As shown in Fig. 1 ▸, the HIREX spectrometer consists of a bent diamond crystal to disperse the transverse beam into energy (Zhu *et al.*, 2012 ▸), and a 1D strip detector.

The glassy carbon spectrometer, alternately, works by dispersing the incoming X-rays in an angle, with a relatively high cross section in the small-angle X-ray scattering (SAXS) region. The scattered X-rays are then collected and reflected by the Si (444) crystal, and collected by a Jungfrau detector (512 × 1024 pixels).

**Figure 2:**
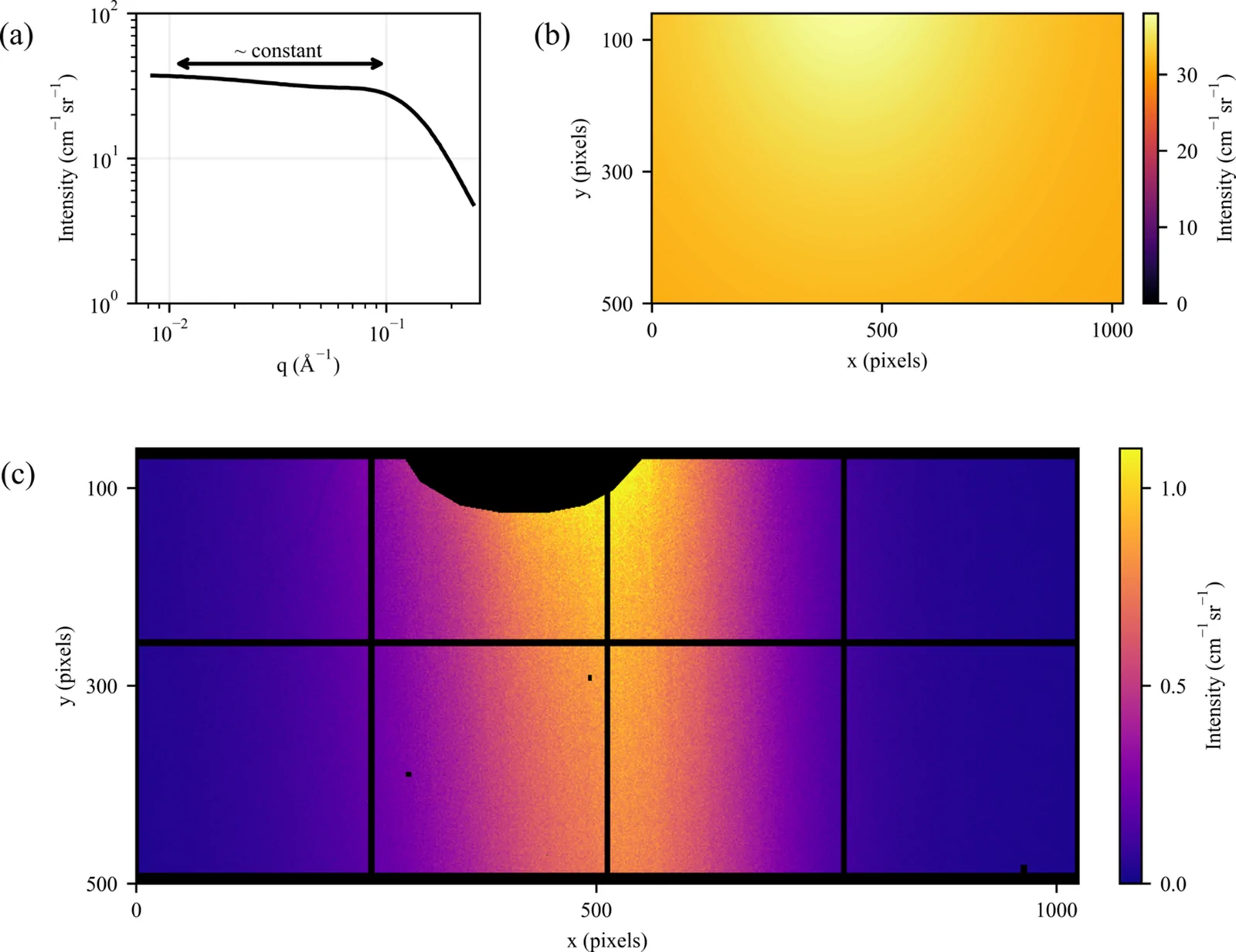
(*a*) Glassy carbon scattering cross section, reproduced from Zhang *et al.* (2010 ▸) under CC BY-NC 2.0. The region of interest corresponds to the approximately flat portion in the range 0.01 Å < *q* < 0.1 Å. (*b*) Glassy carbon correction map for the Jungfrau detector, obtained by mapping the scattering cross-section values onto the corresponding positions on the detector image. (*c*) Jungfrau detector image corrected using the glassy carbon scattering cross section shown in panel (*b*) and masked to exclude the beam block and detector gaps.

A correction map derived from direct SAXS measurements on the glassy carbon (Zhang *et al.*, 2010 ▸) is applied to correct for angle-dependent intensity variations. The map, shown in Fig. 2 ▸(*b*), is applied to the recorded Jungfrau image prior to further analysis, ensuring accurate measurements of the incident spectrum. The corrected detector image, averaged over 11945 shots, is given in Fig. 2 ▸(*c*). The majority of the observed non-uniform intensity is accounted for by this correction, while additional effects (such as per-pixel solid angle and self-attenuation in the glassy carbon) contribute variations within 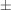
1% of the measured intensity. The broad, relatively flat, dispersion of glassy carbon across a large *q* range, used in tandem with the high-resolution energy selection of the Si (444) crystal, achieves full bandwidth coverage of 50 eV, while maintaining millielectronvolt-scale spectral resolution.

The choice of (*hkl*) values for the flat Si crystal depends on the geometry, as well as the chosen photon energy and the desired spectral range and resolution. The resolution of the spectrometer can be calculated by taking the derivative of the Bragg equation, 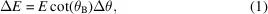
where Δ*E* is the energy bandwidth, *E* is the X-ray photon energy, θ_B_ is the Bragg angle and Δθ is the angular dispersion. The Si (444) is chosen due to its high Bragg angle (72.3°) at the central X-ray energy of 8.31 keV. This enhances the energy dispersion and thereby improves the spectral resolution on the detector. For a perfect flat crystal analyzer, the effective angular spread is the sum of contributions from pixel sampling, finite source size on the glassy carbon, and the intrinsic (Darwin) width of the reflection. Since the pixel size of the Jungfrau detector is 75 µm (Mozzanica *et al.*, 2018 ▸, 2016 ▸), given the geometry depicted in Fig. 1 ▸, the energy-dispersion resolution is 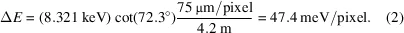
The 4.2 m distance encompasses the glassy carbon to Si distance (3.75 m) and the Si to detector distance (0.45 m). We match the beam size incident on the glassy carbon to the detector pixel size, which leads to equal contributions to the resolution. Therefore, the overall resolution of the glassy carbon spectrometer [including the intrinsic width of the Si (444) reflection, 44.9 meV] is 78.6 meV.

The energy-per-pixel calibration of the glassy carbon spectrum is obtained by taking scans after the insertion of a Si (111) monochromator, which is upstream of the X-ray focusing optics and tuned at energies of −5 eV, 0 eV, +5 eV and +10 eV that are offset from the nominal central energy. A linear fit to the monochromatic scans yields an energy dispersion of 48.1 meV per pixel, approximately matching the expected 47.4 meV per pixel when the combined uncertainties of the monochromator calibration and spectrometer geometry are taken into account.

## 3. Results

**Figure 3:**
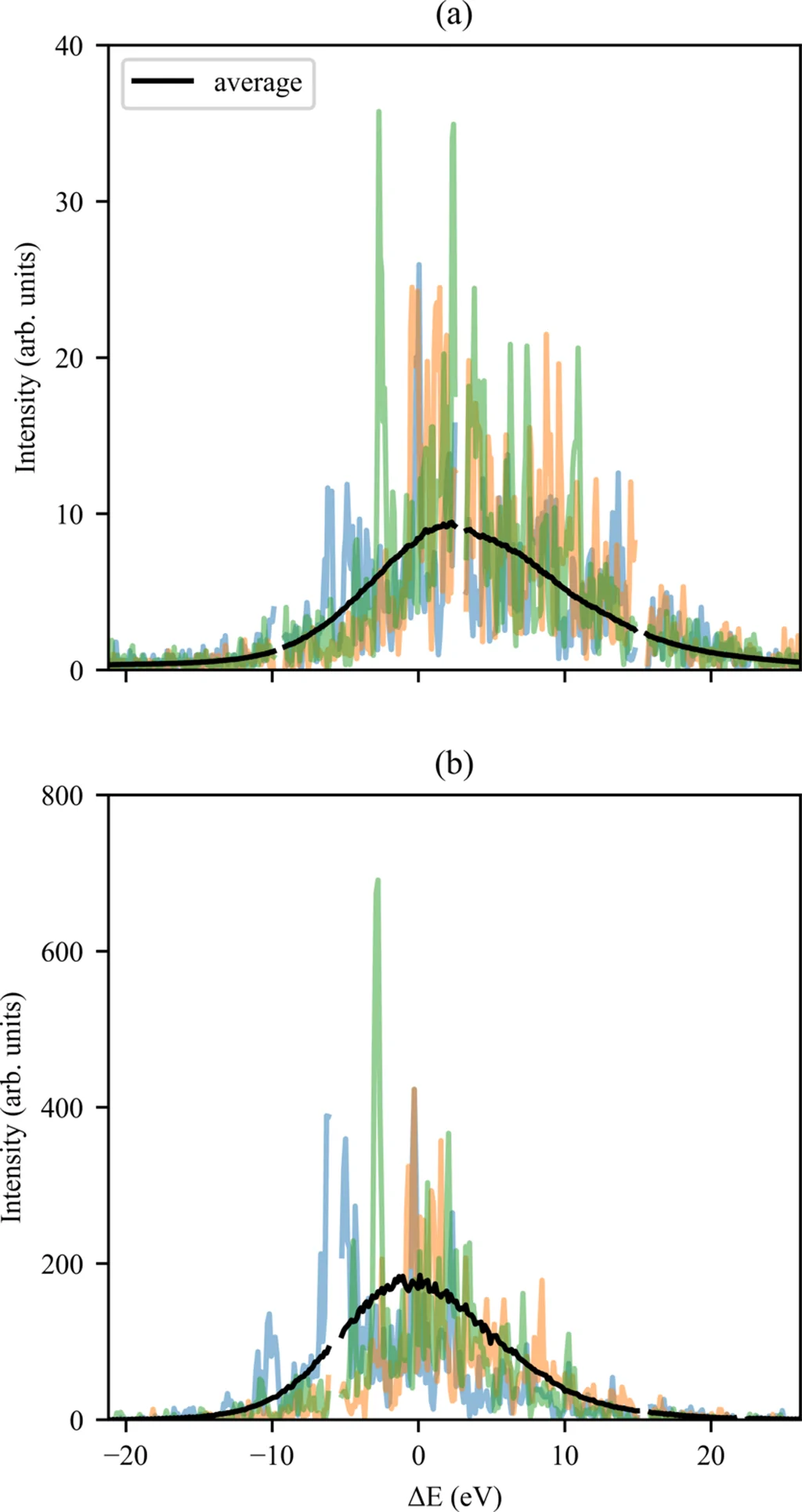
Comparison between the (*a*) glassy carbon and (*b*) HIREX spectrometers. Select single-shot spectra are shown, with an average spectrum (averaged over 9024 shots) drawn in black. The Gaussian FWHM of the SASE spectra recorded by the glassy carbon spectrometer and the HIREX are 15 and 13 eV, respectively.

Examples of single-shot spectra obtained by the glassy carbon spectrometer as well as the HIREX spectrometer are depicted in Fig. 3 ▸. The pulse trains are recorded as train IDs with each spectrum, and, for comparison purposes, only those shots with matching train IDs are considered. Three single-shot spectra are extracted to demonstrate the glassy carbon spectrometer’s capability to take single-shot data, as well as to confirm that our setup has enough resolving power to identify the individual spikes. The average over all valid 9024 shots is overlaid in black. The Gaussian full width at half-maximum (FWHM) of the two independently obtained average spectra are 15 eV as recorded by the glassy carbon spectrometer and 13 eV for the HIREX spectrometer.

### 3.1. SCXS: aluminium plasmon

XRTS is the scattering of X-ray radiation by electrons in a material, which may be free, weakly bound or tightly bound to the ion, producing an X-ray spectrum known as the dynamic structure factor (DSF) (Chihara, 1987 ▸). Forward and backward XRTS spectrometers are used in this study to collect the inelastic scattering corresponding to the collective and non-collective geometries, respectively. The distinction between the collective and non-collective regimes is determined by comparing the spatial scale of the probed density fluctuations, 2π/*q*, with the electron screening length λ_s_ (Glenzer & Redmer, 2009 ▸). This comparison defines the scattering parameter, α = 1/*q*λ_s_, where *q* = 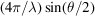 and θ is the scattering angle. In the non-collective regime, which is accessible for large scattering angles (backscattering geometry), α < 1 and the probe length is small compared with the screening length. The scattering of individual electrons can be treated by the impulse approximation (Döppner *et al.*, 2009 ▸) and the scattering signal is dominated by individual electron contributions (Eisenberger & Platzman, 1970 ▸). The collective regime is accessed by small scattering angles (forward-scattering geometry) and α > 1. Here, the probing scale is larger than the screening length, so the scattering radiation is sensitive to collective electron-density oscillations, such as plasmons (Glenzer & Redmer, 2009 ▸; Döppner *et al.*, 2009 ▸). Key plasma parameters like plasma frequency, free electron density and temperature can be directly measured from first principles, provided that the energy-resolved spectrum is measured with sufficient accuracy. Due to the well characterized solid-state behavior of collective plasmon excitations in aluminium, XRTS can be used in this study as a material witness to assess the accuracy of the two incident spectrometers, HIREX and glassy carbon. Here we apply stochastic correlation X-ray spectroscopy (SCXS) to monitor spectral distortions across the full SASE spectrum.

SCXS leverages both the spectral and intensity variation of the SASE X-ray spikes on a shot-to-shot basis while simultaneously providing the necessary photon flux for fast high-resolution data acquisition with high dynamic range. The total scattered spectrum consists of the contribution from an incident SASE pulse spike and the corresponding weighted sum of the elastically and inelastically scattered components. The stochastic nature of the SASE pulses enables the application of a matrix formalism to reconstruct the collective DSF. We define two-dimensional matrices *J*_*km*_ and *S*_*kn*_, composed of the intensity values of the SASE and collective spectrum, respectively. The dimensions correspond to the pulse number (1…*k*), and (1…*m*) and (1…*n*) are the respective energy bins of each spectrometer. The diagonal of the response matrix *R*_*mn*_ = 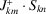 is used to measure the collective DSF, where 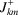 is the Moore–Penrose pseudoinverse matrix (Kayser *et al.*, 2019 ▸).

**Figure 4:**
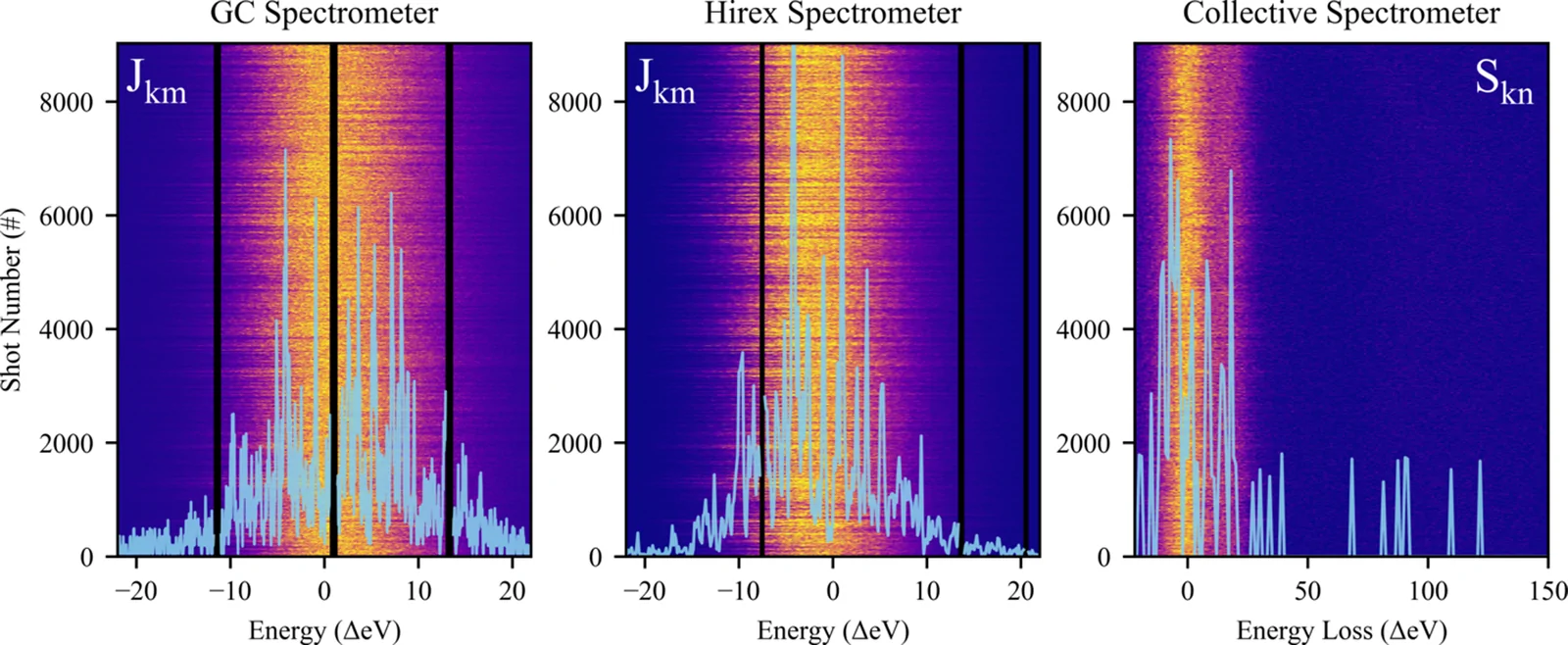
The spectral distributions of incident SASE pulses (glassy carbon and HIREX) and the scattered collective XRTS as a function of shot series, plotted with an example single-shot intensity lineout. Glassy carbon (GC) and HIREX are both represented by *J*_*km*_, with collective XRTS shown as *S*_*kn*_. Detector regions excluded from analysis are indicated in black.

Accurate SCXS analysis is enabled by knowledge of the spectral content of the incident radiation, specifically its intensity and frequency distribution. Because SCXS depends on the relative weighting of individual stochastic spikes within each shot, an accurate characterization of the incident spectrum is essential. Fig. 4 ▸ illustrates the discrepancies observed between the glassy carbon and HIREX measurements of the incident spectrum for a single recorded event. To evaluate which measurement more accurately represents the true spectral distribution, SCXS is applied using each spectrometer’s incident spectrum as an input to the pseudoinverse matrix 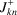.

Aluminium provides an effective benchmark for this comparison because its XRTS response contains a well characterized inelastic plasmon feature that can be reliably modeled (Fletcher *et al.*, 2015 ▸; Gawne *et al.*, 2024 ▸; Preston *et al.*, 2019 ▸). By reconstructing the collective DSF for aluminium under each assumed incident spectrum and comparing the results with the measured scattering signal, SCXS serves as a diagnostic tool for determining which spectrometer, glassy carbon or HIREX, provides the more accurate incident characterization. This approach not only identifies the spectrometer that consistently reproduces the aluminium scattering signal with higher fidelity but also establishes the feasibility of applying SCXS to high energy density (HED) platforms where high-resolution XRTS is a critical diagnostic for determining plasma properties.

### 3.2. Spectral response analysis

**Figure 5:**
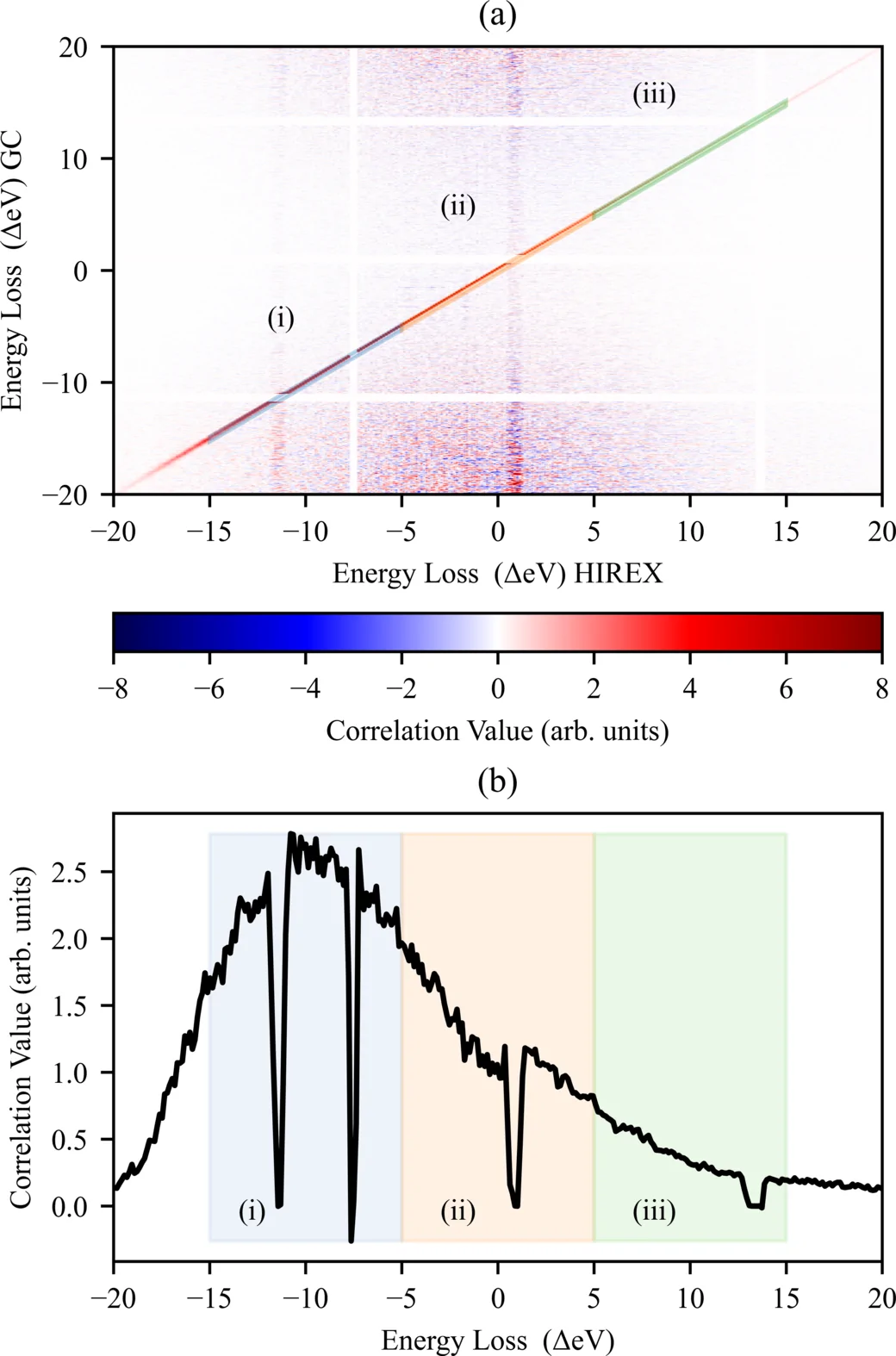
(*a*) The response matrix *R*_*mn*_ of the glassy carbon and HIREX spectrometers with the regions of interest along the line of equal energy highlighted. The white gaps indicate masked detector regions. (*b*) The averaged correlation intensity values along the diagonal of the the response matrix *R*_*mn*_ are plotted and separated into three energy regimes: (i) −15 to −5 eV, (ii) −5 to 5 eV and (iii) 5 to 15 eV.

Differences in spectral performance are evaluated by constructing the response matrix between the glassy carbon spectrometer and HIREX, as shown in Fig. 5 ▸. A correlation intensity is used to partition the spectral response into three distinct energy ranges, enabling identification of spectral regions where the two analyzers diverge.

**Figure 6:**
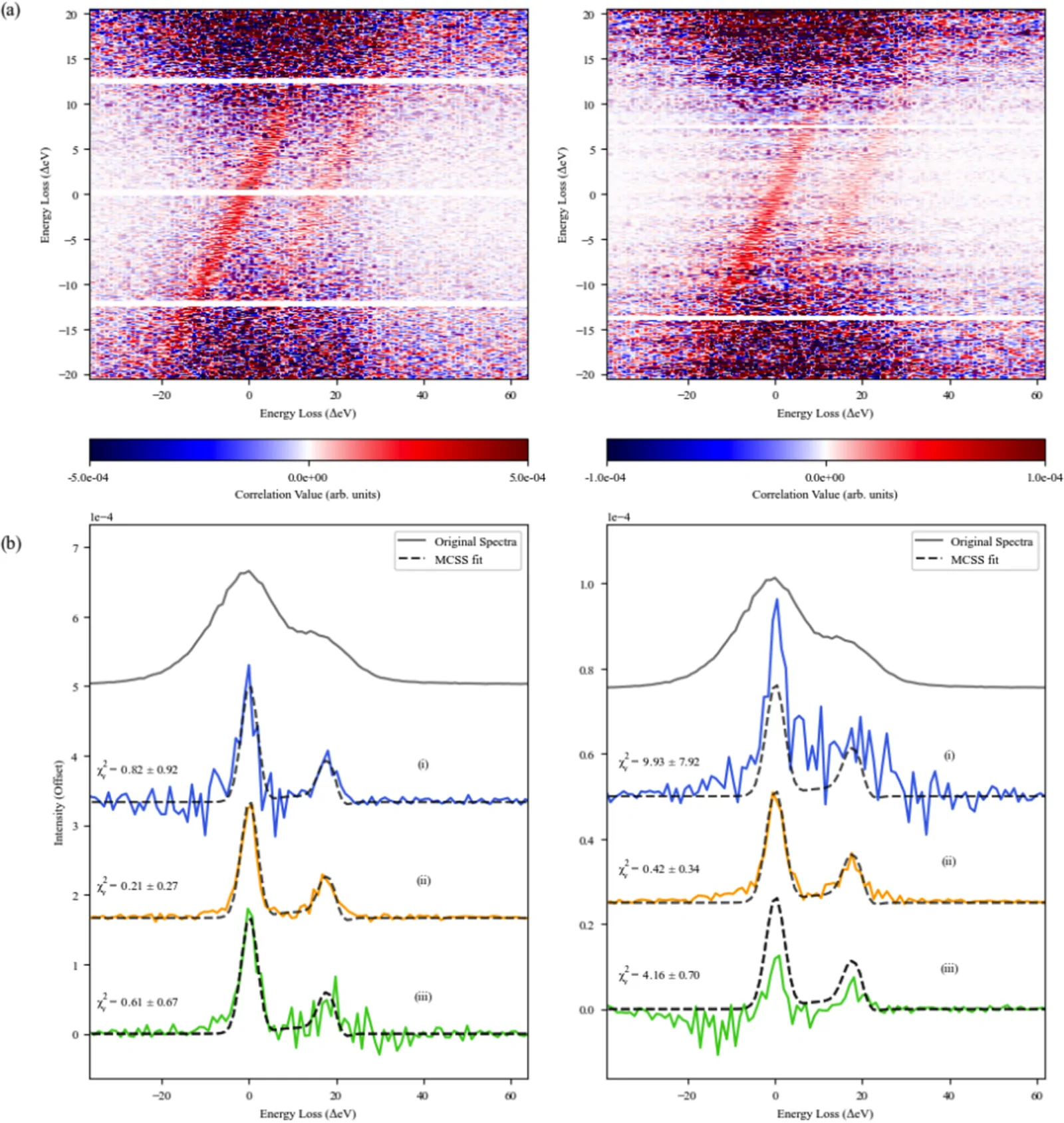
(*a*) Response matrix *R*_*mn*_ for the glassy carbon spectrometer (left) and the HIREX spectrometer (right). (*b*) SCXS results for the three regions of interest, compared with their theoretical XRTS spectra generated using the MCSS code and the original summed XRTS spectra measured from the collective spectrometer.

A total of 9024 individual scattering events from 30 µm-thick aluminium under ambient conditions were analyzed in order to evaluate the spectral performance of the glassy carbon and HIREX spectrometers. Each spectrometer measured the incident SASE matrix, 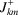, along with the collective spectrometer reference matrix, *S*_*kn*_, as shown in Fig. 4 ▸. From these measurements, a response matrix, *R*_*mn*_, was constructed for each spectrometer, where (1…*m*) corresponds to the energy range of the SASE pulse spectrometer and (1…*n*) corresponds to the energy range of the collective XRTS spectrometer. The spectral deviations arising from sampling the three energy ranges indicated in Fig. 5 ▸ are presented in Fig. 6 ▸.

The theoretical collective DSF expected for aluminium under ambient conditions was calculated using a multi-component scattering simulation (MCSS) (Chapman, 2016 ▸), providing a reference for comparison with the measured response. In this work, we treat the elastically scattered amplitude as an arbitrary parameter normalized to the measured scattered spectrum, which is used here to quantify the intrinsic resolution of our XRTS spectrometer. The inelastic plasmon is modeled for a scattering wavevector of ∼0.95 Å^−1^, well below the Fermi wavevector and the electron–hole pair continuum, where Landau damping can influence the spectral position of the maximum as described by the Bohm–Gross plasmon dispersion relation. The simulation employs an analytic random phase approximation model valid for electron temperatures approaching zero and an electron degeneracy parameter much less than one, corresponding to a non-degenerate (classical) state. The plasma frequency shift is calculated assuming that all three conduction electrons (*Z*_f_ = 3) in aluminium contribute to the inelastic response.

To evaluate the performance of the spectrometers across the different energy regions indicated in Fig. 5 ▸, the measured spectra were summed over three ranges: −15 to −5 eV, −5 to 5 eV and 5 to 15 eV. Each average produces a reconstructed collective DSF, which is compared with the simulated plasmon spectrum. Spectrometer performance is quantified using reduced χ^2^ values, calculated by summing the squared differences between the measured and theoretical plasmon spectra, weighted by the experimental uncertainties. Values less than or equal to 1 indicate that the residual differences are within the estimated uncertainties, implying that the deviations are primarily due to spectrum noise rather than systematic fitting errors.

Within these energy ranges, the HIREX spectrometer exhibits χ^2^ values exceeding 1 for −15 to −5 eV and 5 to 15 eV, whereas the glassy carbon spectrometer maintains χ^2^ values near or below 1 across all three ranges. Specifically, the χ^2^ error for HIREX is 12.11 times larger than that of glassy carbon in the −15 to −5 eV range and 6.82 times larger in the 5 to 15 eV range. These deviations primarily reflect intensity fluctuations above and below unity, as observed in Fig. 5 ▸, and are further evident in the response-matrix reconstruction as broadening and negative values outside the nominal central energy region of −5 to 5 eV. Correlation intensity between the two spectrometers measuring the same SASE beam should remain consistent across all energy ranges if the spectral response was uniform. The correlation intensity is interpreted here as an effective normalization rather than a quantity with unique physical significance. Application of a single global scaling factor yields consistent agreement with the glassy carbon reference over all energies, whereas no single scaling produces comparable agreement with HIREX. This behavior is reflected in the skewed correlation-intensity distribution and indicates that the glassy carbon spectrometer provides a more self-consistent representation of the incident spectrum across the full bandwidth.

The spectrum obtained from the response matrix over the central energy range of −5 to 5 eV is used to evaluate the improvement in the resolving power of the XRTS von Hamos X-ray spectrometer based on highly annealed pyrolytic graphite (HAPG) crystals achieved using the SCXS methodology, compared with the conventional approach of summing the XRTS spectra. SCXS accounts for fluctuations in the incident XFEL spectrum by incorporating the shot-to-shot spectral structure of the incident SASE pulses into the reconstruction. As shown in Fig. 6 ▸(*b*), this reduces the FWHM of the reconstructed collective feature relative to the directly measured shot-averaged forward-scattering spectrum obtained from the HAPG spectrometer. The SCXS method yields a resolving power of *E*/Δ*E* = 2416, corresponding to an improvement by a factor of 5.67 relative to the directly measured spectrum obtained without SCXS reconstruction (gray curve in Fig. 6 ▸), with *E*/Δ*E* = 426, and approaches the intrinsic spectrometer resolving power of *E*/Δ*E* = 2518 (Preston *et al.*, 2020 ▸). Furthermore, the ability to resolve the inelastic feature while preserving the expected intensity supports the application of this method to the investigation of plasma properties in HED experiments, where electronvolt-scale precision is required for accurate measurements.

This work demonstrates a glassy carbon-based spectrometer for shot-to-shot characterization of the incident SASE spectrum in XFEL scattering experiments. By exploiting the spatially uniform scattering response of glassy carbon together with a flat Si (444) crystal, the spectrometer provides an energy-resolved measurement that is insensitive to spatial inhomogeneities, spectral chirp, and central energy jitter of the XFEL beam. Application of the method to collective X-ray scattering from aluminium shows that accurate reconstruction of the collective DSF can be achieved across the entire SASE energy range, with improved agreement with theoretical expectations relative to conventional bent-crystal spectral diagnostics. The results highlight the necessity of this platform for quantitative spectroscopic measurements conducted at XFEL facilities.

## Data Availability

Data recorded for the experiment at the European XFEL will be openly available at https://doi.org/10.22003/XFEL.EU-DATA-008237-00 once the data embargo of the experiment 8237 has been lifted (6 Aug 2027), or available from the corresponding author(s) upon reasonable request.
